# REACH: An innovative model for child eye health

**Published:** 2017

**Authors:** Rahul Ali

**Affiliations:** Country Director, Orbis International, New Delhi, India

**Figure:**
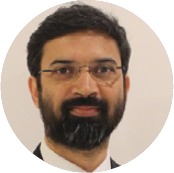
Dr Rahul Ali

**School-based eye health programmes are a golden opportunity to recognise that the timely provision of effective interventions can be a life-changing experience for a child in need.**

**Figure:**
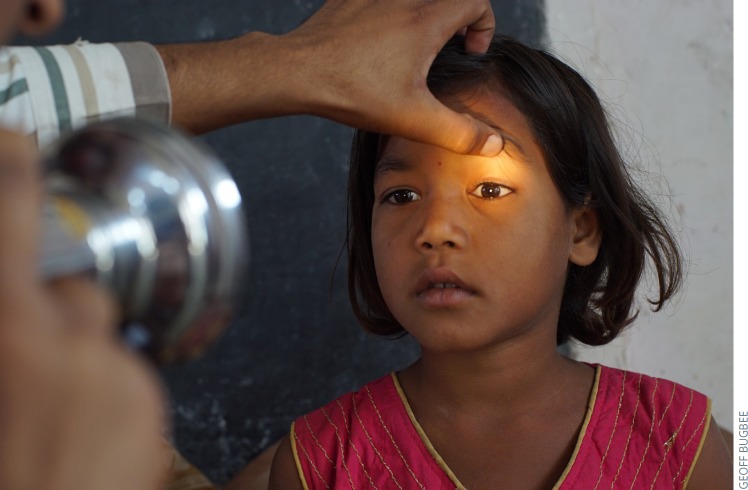
School screening programme. INDIA

School is the first formal space for learning. Using this space to reach the vast cohort of school-aged children who constitute a particularly vulnerable group because of the high prevalence of refractive error is a common practice. There are several models of school eye health programmes currently operational across India. REACH – Refractive Error Among Children is a model aiming to address challenges in the school eye health space and build innovative, sustainable and scalable programmes.

## The REACH model

The REACH model employs a three-phased deployment approach.

### Phase I: Prepare

This phase involves four steps:

- Generate a database of all the schools in the project area
- Establish initial contact with local authorities, schools and other stakeholders to get their buy-in and necessary permissions
- Generate a list of enrolled students in each of the participating schools
- Plan and schedule service delivery activities with an adequate lead time

This phase sets the foundation for subsequent service delivery activities.

### Phase II: Deliver

- The first activity in this phase is primary screening where the vision screener confirms the identity of the child. The screener performs a visual acuity assessment using a backlit pocket vision screener with LogMAR 0.2 optotypes (Snellen 6/9.5). Children who fail the vision screening test or have any other ocular complaints even in one eye or are already wearing spectacles are selected for secondary evaluation.
- During secondary examination, the team which includes an optometrist or ophthalmologist conducts a detailed visual acuity assessment, refraction and ophthalmic evaluation. All children who require spectacles are given a prescription. Three intervention categories may emerge:
- Children identified with simple refractive error may receive spectacles on the spot
- Complex prescriptions that need custom spectacles are delivered at a later date. Nevertheless, children are also given an opportunity to select their frames.
- Children who need cycloplegic refraction may receive it on the spot or are referred to the nearest fixed facility. Any child who may need further evaluation or other intervention is also referred to the base hospital or nearest vision centre for necessary investigation and treatment.
- All children who require spectacles are counseled by a counselor on spectacle care, need for continuous wear of the prescribed spectacles as well as provided information on contacting the concerned person in case of any difficulty, for ensuring acceptance of the dispensed glasses.

In addition to addressing children's eye health needs, the REACH team also screens all teachers and provides orientation on the basics of primary eye care using Orbis' ‘Vision Screening Handbook for School Teachers.’

### Phase III: Consolidate

Often the end-point of school-based programmes is the distribution of spectacles and the entire exercise is a one-off event. It is important to investigate whether children who are given spectacles are using them or not.

**Figure:**
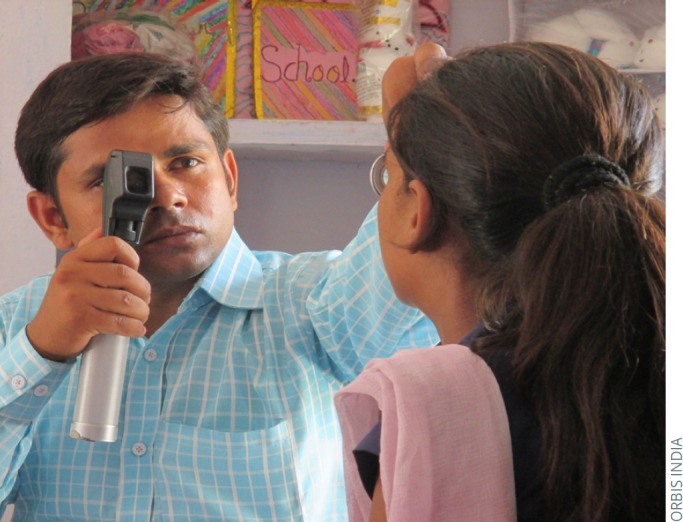
A screener performs a visual acuity assessment using a backlit pocket vision screener. INDIA

In REACH, a team member visits the school unannounced three months after giving glasses to determine compliance and complete a compliance/non-compliance questionnaire. This approach has provided the teams not only with an opportunity to evaluate the success of the intervention but also a chance to identify specific reasons for compliance/non-compliance. The team can also identify children whose glasses need repair or replacement in case of minor problems or breakage of the glasses that were provided earlier.

REACH also has an annual follow-up visit scheduled within the programme. A year later, the same team visits the school to re-evaluate children who underwent a secondary evaluation. New admissions including first grade (elementary), all students in 8th (secondary) and 11th grade (higher secondary) and any students identified by the oriented teachers to have an eye health issue are screened. This visit is planned on an annual basis.

The REACH model is built on four defining features:

## Knowledge Attitude Practice (KAP) study

A KAP study on refractive errors in children amongst children themselves, their parents, teachers and eye health service providers is conducted. The findings of this study guide the development of appropriate Information Education Communication (IEC), training material as well as other awareness generation activities. This creates a strong mechanism to effectively communicate with stakeholders and empower them with the right information to make them receptive and accept the treatment provided. Evidence of any change in eye health seeking behaviour within the community will be documented through a repeat KAP study in the same area.

## Standardisation

Standardisation of process, hardware and software all contribute to making REACH a unique initiative. A common guideline has been developed to standardise both clinical and non-clinical processes. All implementing partners are utilising the same guidelines for vision screening cut-off, spectacle prescription, cycloplegia, referral, compliance evaluation and follow-up, among others. Similarly, key pieces of hardware are standardised across sites, e.g. internally illuminated pocket vision screeners, auto-refractors, LogMAR visual acuity charts as well as child-friendly occluders. And REACHSoft, a bespoke software, is deployed across all sites to manage data at all points of activity within REACH.

## Data Management

To facilitate all of the above and provide good quality information to drive future initiatives, there is a strong focus on data management at all levels and at all steps of REACH. To that end, Orbis has developed REACHSoft, a software solution tailored for this programme. REACHSoft is designed to support the planning, implementation and management (including monitoring and evaluation) of the programme. From the first step of scheduling a visit to a school, REACHSoft supports every step of the planning process and implementation: developing the school database, collecting the school-wise student database, scheduling and planning service delivery activities, collecting data at the individual student level during service delivery (primary screening, detailed examination, spectacle prescription and dispensing, referral management, etc.) as well as monitoring progress and generating reports aiding management of the programme.

## Multi-centric research

While better data management and real-time monitoring facilitates the smooth implementation of the programme, the huge data set which will be developed is intended to be used for multi-centre research. This will in turn provide evidence-based recommendations for improving similar initiatives in the future.

Considering that 80% of what a child learns is visual,^1^ good vision is critical to a child's ability to participate in and benefit from educational experiences. School-based eye health programmes are a golden opportunity to recognise that the timely provision of effective interventions can be a life-changing experience for a child in need. Addressing this need across the country is a mammoth challenge but it is imperative that we REACH out to the millions of children across India.
